# Role of Cryptochrome-1 and Cryptochrome-2 in Aldosterone-Producing Adenomas and Adrenocortical Cells

**DOI:** 10.3390/ijms19061675

**Published:** 2018-06-05

**Authors:** Martina Tetti, Isabella Castellano, Francesca Veneziano, Corrado Magnino, Franco Veglio, Paolo Mulatero, Silvia Monticone

**Affiliations:** 1 Division of Internal Medicine and Hypertension, Department of Medical Sciences, University of Torino, 10126 Torino, Italy; tetti.martina@gmail.com (M.T.); cmagnino@libero.it (C.M.); franco.veglio@unito.it (F.V.); paolo.mulatero@unito.it (P.M.); 2 Division of Pathology, Department of Medical Sciences, University of Torino,10126 Torino, Italy; isabella.castellano@unito.it (I.C.); francesca.veneziano11@gmail.com (F.V.)

**Keywords:** aldosterone-producing adenoma, *CRY1*, *CRY2*, *HSD3B1*, *HSD3B2*

## Abstract

Mice lacking the core-clock components, cryptochrome-1 (CRY1) and cryptochrome-2 (CRY2) display a phenotype of hyperaldosteronism, due to the upregulation of type VI 3β-hydroxyl-steroid dehydrogenase (*Hsd3b6*), the murine counterpart to the human type I 3β-hydroxyl-steroid dehydrogenase (*HSD3B1*) gene. In the present study, we evaluated the role of *CRY1* and *CRY2* genes, and their potential interplay with *HSD3B* isoforms in adrenal pathophysiology in man. Forty-six sporadic aldosterone-producing adenomas (APAs) and 20 paired adrenal samples were included, with the human adrenocortical cells HAC15 used as the in vitro model. In our cohort of sporadic APAs, *CRY1* expression was 1.7-fold [0.75–2.26] higher (*p* = 0.016), while *CRY2* showed a 20% lower expression [0.80, 0.52–1.08] (*p* = 0.04) in APAs when compared with the corresponding adjacent adrenal cortex. Type II 3β-hydroxyl-steroid dehydrogenase (*HSD3B2*) was 317-fold [200–573] more expressed than *HSD3B1*, and is the main *HSD3B* isoform in APAs. Both dehydrogenases were more expressed in APAs when compared with the adjacent cortex (5.7-fold and 3.5-fold, respectively, *p* < 0.001 and *p* = 0.001) and *HSD3B1* was significantly more expressed in APAs composed mainly of zona glomerulosa-like cells. Treatment with angiotensin II (AngII) resulted in a significant upregulation of *CRY1* (1.7 ± 0.25-fold, *p* < 0.001) at 6 h, and downregulation of *CRY2* at 12 h (0.6 ± 0.1-fold, *p* < 0.001), through activation of the AngII type 1 receptor. Independent silencing of *CRY1* and *CRY2* genes in HAC15 cells resulted in a mild upregulation of *HSD3B2* without affecting *HSD3B1* expression. In conclusion, our results support the hypothesis that *CRY1* and *CRY2*, being AngII-regulated genes, and showing a differential expression in APAs when compared with the adjacent adrenal cortex, might be involved in adrenal cell function, and in the regulation of aldosterone production.

## 1. Introduction

Primary aldosteronism (PA), affecting 6% of the general hypertensive population [1], and up to 20% of patients referred to hypertension units [2,3], is widely recognized as the leading cause of endocrine hypertension. Aldosterone-producing adenoma (APA) and bilateral adrenal hyperplasia (BAH) are the most frequent underlying causes of PA, while unilateral adrenal hyperplasia (UAH) is less common. The last few years witnessed major advances in the understanding of the molecular determinants leading to autonomous aldosterone overproduction in both sporadic and familial PA. In particular, the introduction of next-generation sequencing allowed the identification of somatic mutations in four genes differently involved in Ca^2+^ homeostasis (*KCNJ5*, *ATP1A1*, *ATP2B3*, and *CACNA1D*), unraveling the genetic basis of approximately 50% of sporadic APAs [4,5,6,7]. Similarly, new insight was gained from mice lacking the core-clock components, cryptochrome-1 (CRY1) and cryptochrome-2 (CRY2) (*Cry*-null mice) [8]. Mammals, as well as many other organisms including plants, adapt most of their physiologic processes to a 24-h time cycle, generated by an internal molecular oscillator referred to as the circadian clock [9]. At the cellular level, circadian oscillations are generated by a series of genes, whose proteic products form a transcriptional autoregulatory feedback loop, where clock circadian regulator (CLOCK) and aryl hydrocarbon receptor nuclear translocator-like protein 1 (ARNTL, also known as BMAL1) act as positive regulators, while period (PER) and CRY act as negative regulators [10]. *Cry*-null mice displayed salt-sensitive hypertension due to chronic and autonomous aldosterone overproduction by the adrenal glands, as a consequence of the massive upregulation of type VI 3β-hydroxyl-steroid dehydrogenase (*Hsd3b6*), the murine counterpart to the human type I 3β-hydroxyl-steroid dehydrogenase (*HSD3B1*) gene [8]. HSD3B catalyzes the conversion of pregnenolone to progesterone, an enzymatic reaction required for aldosterone biosynthesis [11]; two different *HSD3B* isoforms are expressed in man—*HSD3B1* is mainly expressed in the placenta, while *HSD3B2* localizes primarily in adrenals and gonads [12]. Immunohistochemistry studies in normal human adrenals showed that *HSD3B2* is the predominant isoform, expressed through the zona glomerulosa and the zona fasciculata (ZF), while *HSD3B1* displays faint immunoreactivity, predominantly in the outermost layer zona glomerulosa (ZG) [8,13,14]. Moreover, in APA samples, *HSD3B1* expression was significantly correlated with the expression of the rate-limiting enzyme for aldosterone production—aldosterone synthase (CYP11B2) [15]. Despite much knowledge being gained from the *Cry*-null animal model, the significance of CRY1 and CRY2 in human adrenal function and aldosterone production is still unknown. So far, few reports have investigated the roles of *HSD3B1* and *HSD3B2* in sporadic PA. Therefore, in this study we aimed to (I) evaluate the expressions of *HSD3B1* and *HSD3B2* in a large cohort of 46 adrenal glands, removed from patients in whom a final diagnosis of unilateral PA was achieved; and (II) investigate the expression of *CRY1* and *CRY2* in unilateral sporadic PA, and their roles in aldosterone production in the HAC15 human adrenocortical cell model.

## 2. Results

### 2.1. Expression of CRY1, CRY2, HSD3B1, and HSD3B2 in Adrenal Tissues

**Figure 1:**
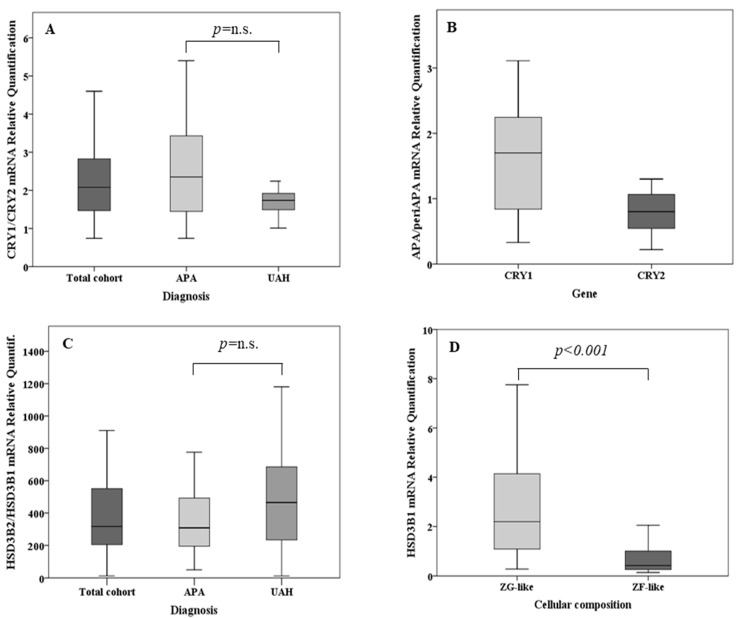

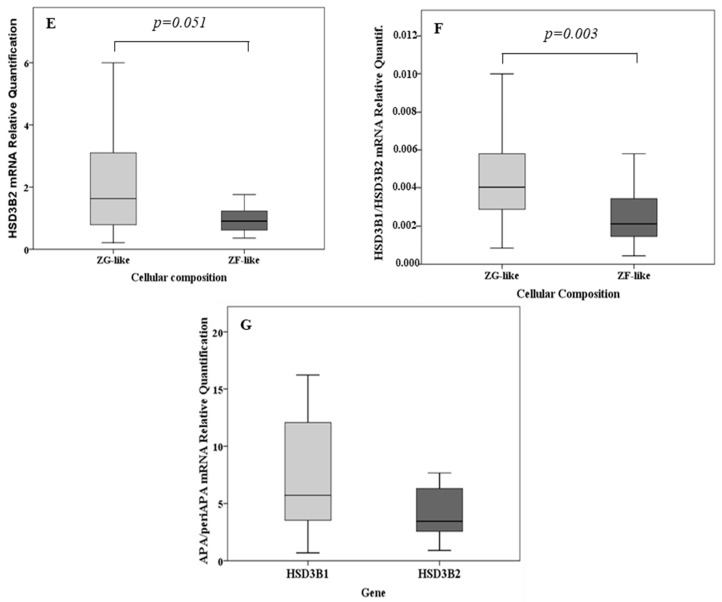
(**A**) Relative quantification of cryptochrome-1 (*CRY1*) mRNA over cryptochrome-2 (*CRY2*) messenger RNA (mRNA) in the total cohort, in aldosterone-producing adenoma (APA) samples (*n* = 35) and in unilateral adrenal hyperplasia (UAH) samples (*n* = 11). (**B**) Relative quantification of *CRY1* and *CRY2* mRNA in APA samples over that in the corresponding adjacent adrenal cortex (*n* = 20). (**C**) Relative quantification of type II 3β-hydroxyl-steroid dehydrogenase (*HSD3B2*) mRNA over type I 3β-hydroxyl-steroid dehydrogenase (*HSD3B1*) mRNA in the total cohort, in APA samples (*n* = 35) and in UAH samples (*n* = 11). (**D**) *HSD3B1* mRNA expression according to the cellular composition in the total cohort of adrenal samples. (**E**) *HSD3B2* mRNA expression according to the cellular composition in the total cohort of adrenal samples. (**F**) Relative quantification of *HSD3B1* mRNA over *HSD3B2* mRNA according to the cellular composition. (**G**) Relative quantification of *HSD3B1* and *HSD3B2* mRNA in APA samples over that in the corresponding adjacent adrenal cortex. For each box plot, the horizontal line represents the median, and the box and bar indicate the 25th to 75th and 5th to 95th percentiles, respectively.

**Figure 2:**
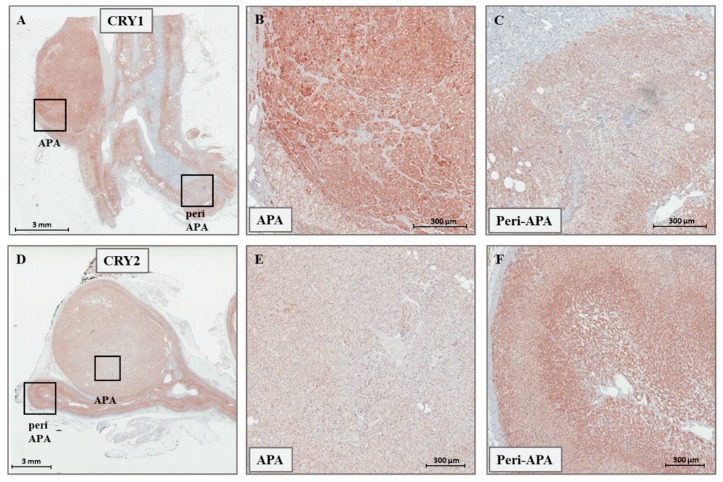
(**A**–**C**) Representative immunohistochemistry staining for *CRY1* in APAs. (**D**–**F**) Representative immunohistochemistry staining for *CRY2* in APAs. Magnifications in (**B**,**C**), and in (**E**,**F**) correspond to the boxed sections in (**A**,**D**), respectively.

The expression levels of *CRY1*, *CRY2*, *HSD3B1*, and *HSD3B2* were determined by real-time PCR in a cohort of 46 sporadic APAs, and 20 paired adjacent adrenal tissues. Within the same sample, the median expression of *CRY1* was 2.1-fold [1.45–2.87] higher than that of *CRY2*, consistently in both APA and UAH (Figure 1A). In our cohort, the expression of both *CRY* genes was neither associated with the cellular composition of the APAs (*CRY1* expression in ZG-like APAs: 1.46 [0.45–2.59], *CRY1* expression in ZF-like APAs: 0.96 [0.49–1.58], *p*-value 0.291; *CRY2* expression in ZG-like APAs: 1.24 [0.57–2.05], *CRY2* expression in ZF-like APAs: 0.84 [0.62–1.28], *p*-value 0.170) nor with the mutational status (*CRY1* expression in wild-type APAs: 1.39 [0.58–2.7], *CRY1* expression in *KCNJ5* mutant APAs: 0.89 [0.42–1.70], *CRY1* expression in *ATP1A1-ATP2B3* mutant APAs: 0.89 [0.49–1.09], *CRY1* expression in *CACNA1D* mutant APAs: 1.4 [0.64–2.61], *p*-value = 0.417; *CRY2* expression in wild-type APAs: 1.08 [0.61–1.98], *CRY2* expression in *KCNJ5* mutant APAs: 0.98 [0.54–1.41], *CRY2* expression in *ATP1A1-ATP2B3* mutant APAs: 0.89 [0.62–1.06], *CRY2* expression in *CACNA1D* mutant APAs: 1.61 [0.80–2.87], *p*-value = 0.170). While the median expression of *CRY1* was 1.7-fold [0.75–2.26] higher in APA tissues when compared with that in the adjacent adrenal cortex (*p* = 0.016), *CRY2* showed a 20% lower expression [0.80, 0.52–1.08] in the nodule when compared with the corresponding surrounding tissue (*p* = 0.04) (Figure 1B). Representative immunohistochemistry staining of frozen tissue sections showing the expression of *CRY1* and *CRY2* in APA and adjacent adrenal cortex is illustrated in Figure 2A–F. 

**Figure 3:**
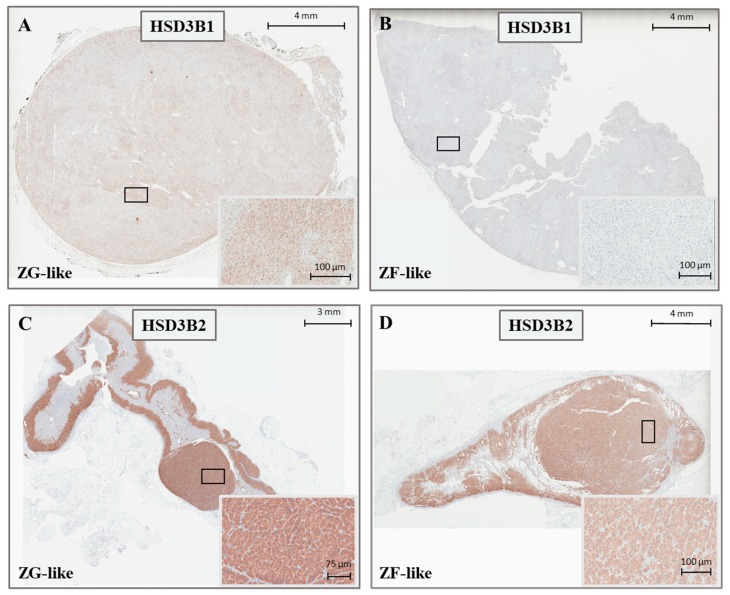
Representative immunohistochemistry staining for *HSD3B1* (**A**,**B**) and *HSD3B2* (**C**,**D**) in APAs, according to the cellular composition.

In both APA and UAH samples, *HSD3B2* was the main isoform, with an overall median expression 317-fold [200–573] higher than that of *HSD3B1* (*p* < 0.001) (Figure 1C). *HSD3B1* transcription was significantly more abundant (median fold change 5.2, *p* < 0.001) in APAs that were composed mainly of ZG-like cells when compared with APAs that had a ZF-like morphology (Figure 1D). A tendency towards a higher *HSD3B2* expression in APAs composed mainly of ZG-like cells was observed, but the difference did not reach statistical significance (median fold change 1.8, *p* = 0.051) (Figure 1E). In addition, the median *HSD3B1*/*HSD3B2* relative ratio was 1.9-fold higher in APA samples composed mainly of ZG-like cells (*p* = 0.003) when compared with APAs composed mainly of ZF-like cells (Figure 1F). No differences in the expression of *HSD3B1* or *HSD3B2*, according to the mutational status (*HSD3B1* expression in wild-type APAs: 1.37 [0.42–4.5], *HSD3B1* expression in *KCNJ5* mutant APAs: 0.51 [0.30–1.97], *HSD3B1* expression in *ATP1A1-ATP2B3* mutant APAs: 0.66 [0.27–1.63], *HSD3B1* expression in *CACNA1D* mutant APAs: 2.27 [1.40–3.12], *p*-value = 0.212; *HSD3B2* expression in wild-type APAs: 1.0 [0.75–2.37], *HSD3B2* expression in *KCNJ5* mutant APAs: 1.01 [0.68–2.18], *HSD3B2* expression in *ATP1A1-ATP2B3* mutant APAs: 0.69 [0.46–0.93], *HSD3B2* expression in *CACNA1D* mutant APAs: 2.82 [1.16–3.19], *p*-value = 0.147) or the final diagnosis (*HSD3B1* expression in APAs: 1.37 [0.30–3.00], *HSD3B1* expression in UAHs: 0.66 [0.42–3.61], *p*-value 0.899; *HSD3B2* expression in APAs: 1.17 [0.66–2.63], *HSD3B2* expression in UAHs: 0.99 [0.71–1.63], *p*-value 0.523), were observed. Notably, both *HSD3B1* and *HSD3B2* were significantly more expressed in the main nodule when compared with adjacent adrenal tissue (5.7- and 3.5-fold, respectively, *p* < 0.001 and *p* = 0.001) (Figure 1G). Representative immunohistochemistry staining of frozen tissue sections showing the expression of *HSD3B1* and *HSD3B2* in APAs, according to the cellular composition, is illustrated in Figure 3A–D.

### 2.2. Regulation of CRY1, CRY2, HSD3B1, and HSD3B2 Expression in HAC15 Cells

To investigate the potential roles of *CRY1* and *CRY2* genes in adrenal cell function and aldosterone production, we used HAC15 adrenocortical cells as an in vitro model. *CRY1* and *CRY2* genes were transcribed in HAC15 cells to a level comparable to that of a pooled set of APA samples, while *HSD3B2* was 35-fold (25–61, *p* < 0.001) more expressed than *HSD3B1*.

HAC15 cells, which were previously reported to express the AngII type 1 receptor [16], were stimulated with AngII (±1 µM irbesartan) or forskolin for 6, 12, and 24 h, and were then harvested for RNA extraction and gene-expression studies.

As expected, treatment with AngII (100 nM) resulted in a significant increase in *CYP11B2* expression at 12 h (68 ± 20-fold over basal, *p* < 0.001). 

**Figure 4:**
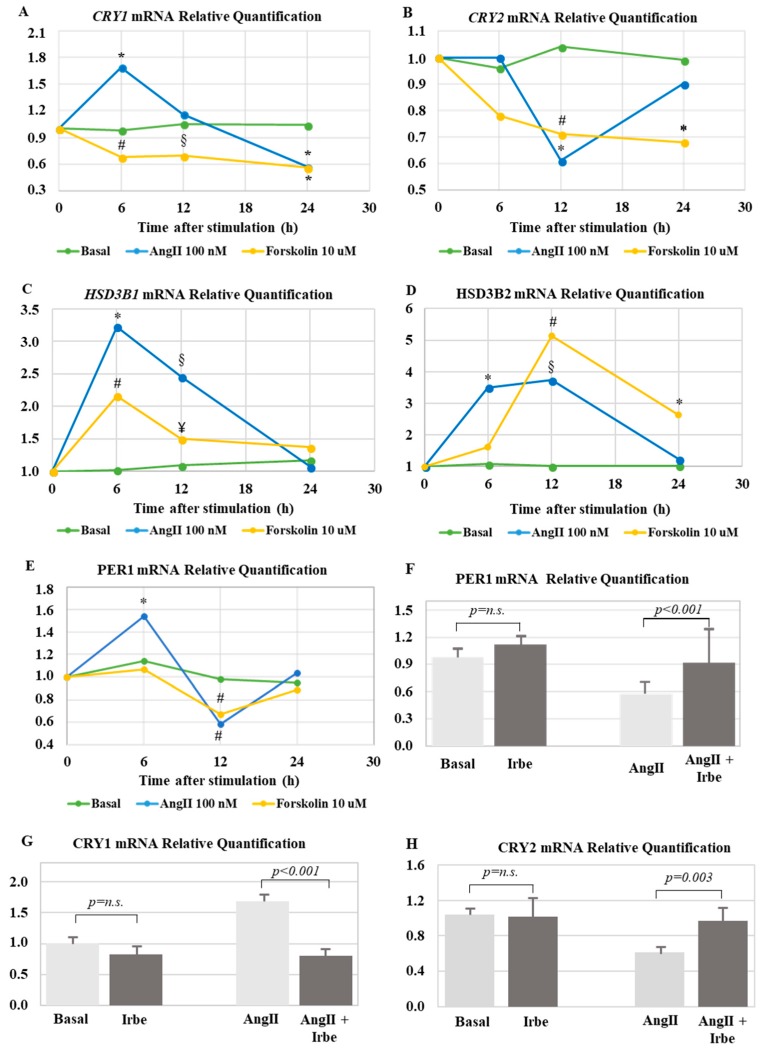
(**A**) Real-time PCR analysis of *CRY1* gene expression. * *p*-Value < 0.001, ^#^ *p*-value = 0.007, and ^§^ *p*-value = 0.017 when compared with basal. (**B**) Real-time PCR analysis of *CRY2* gene expression. * *p*-value < 0.001 and ^#^ *p*-value = 0.003 when compared with basal. (**C**) Real-time PCR analysis of *HSD3B1* gene expression. * *p*-value = 0.022, ^#^ *p*-value = 0.03, ^§^ *p*-value < 0.001, and ^¥^ *p*-value = 0.023 when compared with basal. (**D**) Real-time PCR analysis of *HSD3B2* gene expression. * *p*-value < 0.001, ^#^ *p*-value = 0.001, and ^§^ *p*-value = 0.009 when compared with basal. (**E**) Real-time PCR analysis of period (*PER1*) gene expression. * *p*-value = 0.001 and ^#^ *p*-value < 0.001 when compared with basal. (**A**–**E**) Each point expresses the mean fold change over basal expression in at least three independent experiments. (**F**) Real-time PCR analysis of *PER1* gene expression at 6 h, after stimulation with 100 nM AngII ± 1 µM irbesartan. (**G**) Real-time PCR analysis of *CRY1* gene expression at 6 h, after stimulation with 100 nM AngII ± 1 µM irbesartan. (**H**) Real-time PCR analysis of *CRY2* gene expression at 12 h, after stimulation with 100 nM AngII ± 1 µM irbesartan. (**F**–**H**) Each bar represents the mean ± SD of relative fold change of gene expression in three independent experiments.

Treatment with AngII significantly increased the expression of *CRY1* mRNA within 6 h (1.7 ± 0.25-fold, *p* < 0.001). Following a peak in expression, the levels of *CRY1* mRNA returned to basal levels after 12 h of AngII treatment (Figure 4A). With respect to *CRY2* expression, stimulation with AngII resulted in a significant downregulation (0.6 ± 0.1-fold, *p* < 0.001) at 12 h (Figure 4B), followed by a return to basal levels at 24 h.

Treatment with forskolin, which mimics adrenocorticotropin (ACTH)-mediated elevation of intracellular cyclic adenosine monophosphate (cAMP), resulted in a downregulation of *CRY1* at 6, 12, and 24 h, and a downregulation of *CRY2* and at 12 and 24 h (Figure 4A,B).

Additionally, AngII and forskolin treatment positively regulated the transcription of both *HSD3B1* and *HSD3B2*. Following a 6-h stimulation with AngII, we observed that *HSD3B1* was 3.2 ± 2.4-fold (*p* = 0.035) more expressed when compared with basal conditions, while the maximum upregulation of *HSD3B2* was observed at 12 h (3.7 ± 0.4-fold, *p* = 0.002) (Figure 4C,D). Similarly, forskolin treatment induced a significant upregulation of both *HSD3B1*, with a peak at 6 h, and *HSD3B2*, with a peak at 12 h, (2.1 ± 1.2-fold and 5.1 ± 2.1-fold, *p* = 0.03 and *p* = 0.001, respectively).

Consistently, after 6 h of AngII stimulation, we detected a 1.5 ± 0.2-fold upregulation of *PER1*, that acts as a negative regulator of the core clock together with *CRY*, followed by a 42% reduction at 12 h, when compared with basal levels (Figure 4E).

Pre-treatment with irbersartan (1 µM) reverted the effects of AngII on *PER1*, *CRY1*, and *CRY2* expression (Figure 4F–H), indicating that the observed effects on gene expression were mediated by the activation of the AngII type 1 receptor.

### 2.3. Effect of CRY1 and CRY2 Silencing in HAC15 Cells

Our observation of the regulation of *CRY* genes by AngII, together with the experimental evidence available from Cry-null mice [8], prompted us to investigate the effect of *CRY* silencing on gene expression in HAC15 cells.

**Figure 5:**
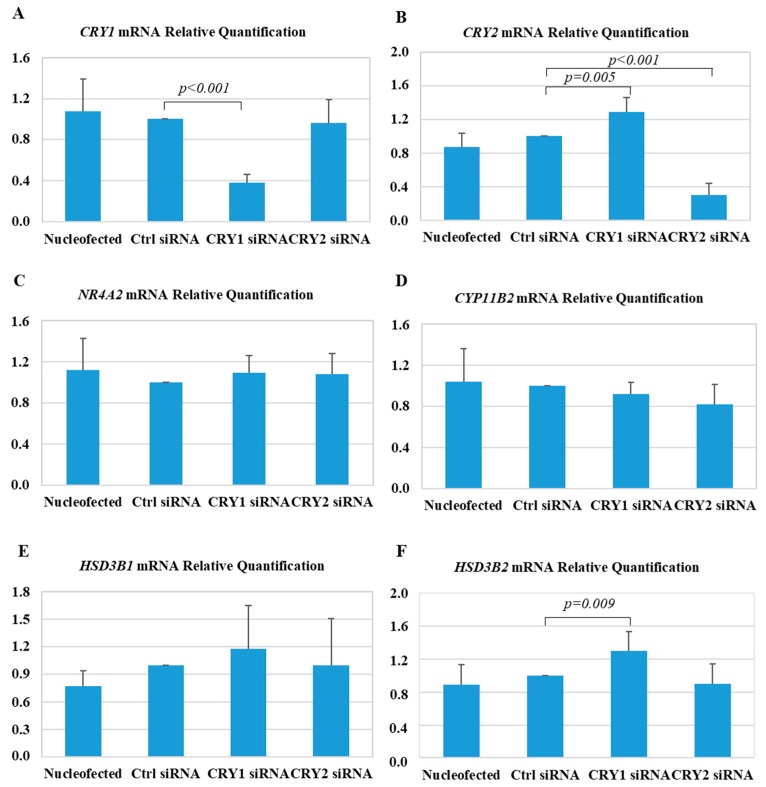
Effect of silencing *CRY1* and *CRY2* on gene expression in HAC15 adrenocortical cells. Real-time PCR analysis of *CRY1* (**A**), *CRY2* (**B**), *HSD3B1* (**C**), *HSD3B2* (**D**), *NR4A2* (**E**), and *CYP11B2* (**F**) gene expression. Each bar expresses the mean ± SD fold change over the expression in cells transfected with a control small interfering RNA (siRNA; Ctrl siRNA) of at least five independent experiments. No significant differences were detected between the cells transfected with Ctrl siRNA, and electroporated cells (Nucleofected).

Silencing *CRY* genes by transfection of siRNA resulted in a 62% reduction in *CRY1* mRNA levels, and a 70% reduction in *CRY2* mRNA levels, measured by real-time PCR (Figure 5A,B). Notably, silencing *CRY1* induced a significant upregulation of *CRY2* (1.3 ± 0.2-fold, *p* = 0.005) (Figure 5B), which resulted in less efficient *CRY2* silencing when the double *CRY1* and *CRY2* siRNA assay was performed, for this reason simultaneous silencing of both genes was not allowed.

The expression of mRNA-encoding key enzymes involved in the production of aldosterone was examined. Transfection with *CRY1* siRNA resulted in a significant upregulation of *HSD3B2* expression (1.30 ± 0.23-fold, *p* = 0.009) (Figure 5F), and a trend toward the upregulation of *HSD3B1* (1.20 ± 0.5-fold, *p* = not significant) (Figure 5E), while the transfection with *CRY2* siRNA did not affect the expression of either *HSD3B1* or *HSD3B2*. Similarly, the expression of *CYP11B2*, and its main transcriptional factor *NR4A2* were not significantly modified at the evaluated timepoint (42 h post-transfection) (Figure 5C,D).

## 3. Discussion

Over the last few years, significant knowledge about the molecular mechanisms that regulate aldosterone overproduction was gained from both next-generation sequencing studies [17], and murine models of primary aldosteronism [18]. The *Cry*-null mice, lacking the core-clock components CRY1 and CRY2 [8], displayed hyperaldosteronism and salt-sensitive hypertension, most likely sustained by the upregulation of the type VI 3β-hydroxyl-steroid dehydrogenase (*Hsd3b6*), corresponding to the human type I 3β-hydroxyl-steroid dehydrogenase (*HSD3B1*) gene.

Immunohistochemistry studies in normal human adrenals showed that HSD3B2 was the predominant isoform, while HSD3B1 localized mainly in the outermost layer zona glomerulosa [8,13,14]. In adrenal pathology, HSD3B1 appeared to be strongly expressed in the hyperplastic zona glomerulosa cells of BAH samples, while its expression was low in a series of eight APAs, composed predominantly of zona fasciculata-like cells [13]. Based on these results, it was hypothesized that HSD3B1 overexpression might represent the molecular mechanism responsible for autonomous aldosterone overproduction in BAH [19].

So far, the role and clinical significance of *CRY1* and *CRY2* genes in the regulation of aldosterone production and APA development, together with their potential interplay with *HSD3B* isoforms, were not explored in humans.

In this study, we demonstrated, for the first time, that *CRY1* is overexpressed, while *CRY2* is downregulated in APA tissue, when compared with the paired adjacent adrenal cortex, which represents the optimal control tissue, given the multiplicity of factors that influence the transcription of the core-clock genes [9]. In agreement with previous reports [15], we observed that *HSD3B2*, being over 300-fold more expressed than *HSD3B1*, is the principal isoform in APAs. A previous study showed that HSD3B1 (evaluated as H-score) was more expressed in APAs carrying somatic mutations in the *KCNJ5* gene [15], while in our cohort we did not detect any significant association between the expression of *HSD3B1* (evaluated by real-time PCR) and the mutational status of the samples. On the contrary, we observed that both *HSD3B1* expression and the relative *HSD3B1*/*HSD3B2* ratio were significantly more elevated in APAs composed mainly of zona glomerulosa-like cells (while APAs carrying a mutation in *KCNJ5* are composed mainly of zona fasciculata-like cells [20]). 

Additionally, this study demonstrated, for the first time, that the expression of both *CRY1* and *CRY2* genes is modulated by AngII through activation of the AT1R. Similarly, the negative regulator *PER1* showed an AngII-dependent regulation. It was previously reported that stimulation with AngII for three hours induced the negative regulator of the core-clock protein PER1 in H295R adrenocortical cells [21]. Additionally, overexpressing *PER1* in H295R cells was able to induce CYP11B1 and CYP11B2 promoter activity [21]. A role for the circadian-clock protein PER1 in the regulation of aldosterone production was recently reported in both in vitro and in vivo studies. *Per1* knock-out mice displayed lower aldosterone levels when compared with wild-type animals, and also a lower expression of *Hsd3b6* in adrenal gland tissue [22]. Silencing *PER1* in H295R cells was able to decrease the expression of *HSD3B1* isoform by 58%, supporting the hypothesis that *PER1* is involved in the modulation of serum aldosterone levels [22].

In the presented study, we showed that AngII stimulation triggers the expression of both *HSD3B1* and *HSD3B2* in HAC15 cells; our results differ from those reported by Ota T. et al. [23], showing that AngII can induce the expression of *HSD3B1*, but not *HSD3B2* in H295R cells. 

To further investigate the potential role of *CRY1* and *CRY2* in the regulation of *HD3B* isoforms, we transfected HAC15 cells with *CRY1* and *CRY2* siRNA. Contrary to what was expected from the *Cry*-null and the *Per* knock-out murine models, silencing *CRY* genes did not modify the expression of *HSD3B1*; however, we observed a mild upregulation of *HSD3B2* in HAC15 cells transfected with *CRY1* siRNA. However, as previously described [24], silencing *CRY1* resulted in a significant upregulation of *CRY2*, which did not allow us to perform an efficient double silencing, and could, therefore, have affected the results, representing a limitation of the presented study.

## 4. Materials and Methods

### 4.1. Patients Selection

A total of 46 adrenal adenomas and 20 paired adjacent adrenal samples were included in the presented study. The adrenal glands were removed from patients affected by unilateral PA, diagnosed in our tertiary referral hypertension centre (Division of Internal Medicine 4—Hypertension Unit, at the University of Torino, Italy). The diagnostic work-up for PA was performed according to the recommendations of the Endocrine Society clinical practice guideline [25]. After withdrawal of interfering medications, the ratio of aldosterone to plasma renin activity was used as a screening test for PA. To confirm the diagnosis, either an intravenous (i.v.) saline load test or a captopril challenge test (when acute plasma volume expansion was contraindicated) was performed. All patients with confirmed PA underwent adrenal computed tomography (CT) scanning and adrenal vein sampling (AVS), as previously described [26]. All patients showing lateralization upon AVS underwent unilateral laparoscopic adrenalectomy. The diagnosis of unilateral PA was confirmed based on clinical benefit and a complete biochemical outcome after adrenalectomy, as defined according to a recent consensus (Primary Aldosteronism Surgical Outcome, PASO) [27]. Clinical and biochemical parameters (before and after adrenalectomy) of the included patients are summarized in Table S1. Normal adrenal glands were obtained from normotensive patients who underwent unilateral nephrectomy for renal carcinoma. For all samples, any adrenal gland showing involvement in the tumor lesion was excluded upon histological examination. All patients gave their written informed consent for the use of samples and clinical data, and the protocol of the study was approved by our local ethics committee, (Comitato etico interaziendale A.O.U. Città della Salute e della Scienza di Torino), Project ID CEI/28, date of approval 14 May 2007).

### 4.2. DNA Sequencing for KCNJ5, ATP1A1, ATP2B3, and CACNA1D

DNA fragments from *KCNJ5*, *ATP1A1*, *ATP2B3*, and *CACNA1D* were amplified by PCR as previously reported [28,29]. The validity of novel mutations was confirmed by sequencing both strands of an independently amplified PCR fragment. Of the presented cohort of adrenal samples, 36 were included in the study by Fernandes-Rosa et al. [29]. Of the included samples, 16 adrenal nodules carried a mutation in the *KCNJ5* gene, six in the *ATP1A1* or *ATP2B3* genes, and five in the *CACNA1D* gene, while 19 samples had no mutations in any of these genes.

### 4.3. Pathological Analysis

Histological examination was performed by an experienced pathologist (I.C.). All adrenal glands included in the study were embedded in paraffin, cut into 3-µm-thick slices, and stained with hematoxylin and eosin (H&E). After accurate macroscopical and microscopical analysis, the final diagnosis of APA was established when a single nodule was present, while the final diagnosis of unilateral adrenal hyperplasia (UAH) was established in the presence of several nodules of varying sizes (with or without a dominant one). In the case of UAH, the dominant nodule was used for gene-expression studies, provided that it was the one identified with *CYP11B2* expression upon immunohistochemistry analysis.

After examination for the known features of ZF (large, lipid-laden clear cells with round to oval vesicular nuclei), ZG (small, compact cells with a high nuclear/cytoplasmic ratio, and a moderate amount of lipids), and zona reticularis (lipid-sparse cytoplasm, and compact cells) cells [20,30], the tumors were categorized as ZF-like when the percentage of large vacuolated cells was greater than 50%, and ZG-type when the percentage of ZF-like cells was ≤50% and the ZG-like cells were the prevalent cell type. Of the analyzed samples, 36 were previously included in the study by Monticone et al. [31].

The final histopathological diagnosis was APA in 35 cases, and multinodular hyperplasia in 11 cases. Twenty-five samples (23 APA samples and two UAH samples) were composed mainly of ZG-like cells, and 21 samples (19 APA samples and 3 UAH samples) were composed mainly of ZF-like cells.

### 4.4. Immunohistochemistry Analyses

Immunohistochemistry analysis was performed using the following primary antibodies: CYP11B2 (CYP11B2-41-17) [32], HSD3B1 (Abnova), HSD3B2 (KAL­KG619) [13], Cry1 (Abgent), and Cry2 (Abcam), as detailed in Table S2.

Formalin-fixed paraffin-embedded tumor samples were cut into sequential 2-μm-thick sections, and deparaffinized and stained at the Pathology Department using a fully automated Ventana BenchMark ULTRA stainer (Ventana, Tucson, AZ, USA), according to the manufacturers’ instructions. Binding of peroxidase-coupled antibodies was detected using the ultraView Universal DAB Detection Kit as a substrate, and the sections were counterstained with hematoxylin.

### 4.5. Cell Culture and Transfection

HAC15 adrenocortical cells were cultured as previously reported [33]. For experiments, cells were plated at a density of 4 × 10^5^ cells/well in a 12-well plate for 48 h. After overnight incubation in low-serum medium (DMEM/F-12 containing 0.1% cosmic calf serum, and antibiotics), cells were stimulated with 100 nM AngII (reference value in normotensive individuals 24 ± 17 pM [34]) (Sigma #A9525) ± 1 µM irbesartan (Sigma #I2286) or forskolin (10 µM, Sigma #F6886) for 6, 12, and 24 h, and then harvested for RNA extraction, and gene-expression studies. 

*CRY1* and *CRY2* gene silencing was performed using the Amaxa technology (Program X-005). One million cells were electroporated in 100 µL of Nucleofector solution R, using 2 µL of a 100-µmol/L solution of Silencer Select predesigned small interfering RNA (siRNA) (Thermo Fisher Scientific, Waltham, MA, USA). After electroporation, cells were plated in a six-well plate, and recovered for 24 h. The medium was then changed to experimental low-serum medium (0.1% cosmic calf serum), and the cells were starved overnight. The following morning, cells were harvested for RNA extraction, and gene-expression studies.

### 4.6. RNA Extraction, and Gene-Expression Studies

RNA isolation from adrenal tissue and cultured cells, and subsequent reverse transcription were both performed as previously reported [33]. Real-time PCR was performed in triplicate using TaqMan gene-expression assays (Thermo Scientific) for *CRY1*, *CRY2*, *HSD3B1*, *HSD3B2*, *PER1*, *NR4A2*, and *CYP11B2*. Gene-expression levels were analyzed using the 2^−ΔΔ*C*t^ relative quantification method, using 18S RNA or *GAPDH* as endogenous reference genes.

### 4.7. Statistical Analyses

IBM SPSS statistics v. 24 was used for the statistical analysis. Data were expressed as mean ± standard deviation or median [25°–75°]. Differences between variables were evaluated using one-way ANOVA followed by Bonferroni’s or Dunnett’s post-hoc tests, when appropriate, and paired (to compare the expression between the adrenal nodule and the corresponding adjacent adrenal cortex) or unpaired *t*-tests or Mann–Whitney tests. A probability of less than 0.05 was considered as statistically significant.

## 5. Conclusions

Our results supported the hypothesis that *CRY1* and *CRY2*, being AngII-regulated genes, and showing a differential expression in APAs when compared with the adjacent adrenal cortex, might be involved in adrenal cell function, and in the regulation of aldosterone production. However, silencing *CRY1* and *CRY2* expression in HAC15 adrenocortical cells resulted only in a modest upregulation of the *HSD3B2* gene, which was not consistent with the experimental observations in the *Cry*-null animal model. Species differences should be considered when studying the role of these genes in adrenal function, and further exploration in this research area is warranted to elucidate the complex role of the circadian clock in adrenal aldosterone production.

## Supplementary Materials

Supplementary materials can be found at http://www.mdpi.com/1422-0067/19/6/1675/s1.
