# Supplementary material for: Role of Cryptochrome-1 and Cryptochrome-2 in Aldosterone-Producing Adenomas and Adrenocortical Cells
Source: Int J Mol Sci. 2018 Jun 5;19(6):1675. doi: 10.3390/ijms19061675 (PMC6032245; doi:10.3390/ijms19061675)
Supplement: Supplementary file 1 [file ijms-19-01675-s001.pdf]

Role of cryptochrome-1 and cryptochrome-2 in aldosterone producing adenomas and adrenocortical cells

Martina Tetti, Isabella Castellano, Francesca Veneziano, Corrado Magnino, Franco Veglio, Paolo Mulatero, Silvia Monticone.

Affiliations

Division of Internal Medicine and Hypertension (M.T., S.M., C.M., F.Veglio, P.M.) and Pathology (I.C., F.Veneziano) Department of Medical Sciences, University of Torino, Torino, Italy.

Short title: Chriptocromes expression in aldosterone producing adenomas

Key words: aldosterone producing adenoma, CRY1, CRY2, HSD3B1, HSD3B2

Abbreviations: PA: primary aldosteronism; APA: aldosterone producing adenoma; BAH: bilateral adrenal hyperplasia; AVS: adrenal vein sampling

Word count: abstract 268 words, manuscript: 3161 words

Number of figures/tables: 5 figures (in colour), and 1 supplemental file.

Corresponding author and person to whom reprints should be addressed:

Silvia Monticone, Department of Medical Sciences, Division of Internal Medicine and Hypertension, University of Torino, Via Genova 3, 10126, Torino, Italy.

Phone: +39-0116336959

E-mail address: silvia.monticone@unito.it

Authors disclosure/conflict of interest: the authors have nothing to disclose.

**Parameter**	**Total (n=46)**	**APA (n=35)**	**UAH (n=11)**	**P-value (APA vs UAH)**
Sex (M/F)	29/17	21/14	8/3	n.s.
Age (years)	48±11	47±10	50±13	n.s.
**Pre-operative**				
SBP (mmHg)	165±25	167±28	158±14	n.s.
DBP (mmHg)	102±13	103±13	98±12	n.s.
Aldosterone (ng/dL)	45 [30-52]	45 [30-52]	46 [32-68]	n.s.
PRA (ng/nl/h)	0.3 [0.2-0.5]	0.3 [0.2-0.5]	0.3 [0.2-0.4]	n.s.
Serum K^+ (mmol/L)	2.9±0,5	2.9±0,5	3.1±0,4	n.s.
Drug number	3 [2-3]	3 [2-3]	2.5 [1-3]	n.s.
**Post-operative**				
SBP (mmHg)	127±11	126±11	130±12	n.s.
DBP (mmHg)	79±7	79±7	80±5	n.s.
Aldosterone (ng/dL)	9 [6-12]	9 [6-13]	9 [4-11]	n.s.
PRA (ng/nl/h)	2.1 [0.6-2.9]	2.1 [1.0-1.3]	1.7 [0.5-3.4]	n.s.
Serum K^+ (mmol/L)	4.8±0.4	4.8±0.5	4.7±0.3	n.s.
Drug number	0.5 [0-1]	0 [0-1]	1 [0-2]	n.s.

Supplemental Table S1. Pre-operative and post-operative clinical and biochemical parameters of included patients. APA = aldosterone producing adenoma; UAH = unilateral adrenal hyperplasia; n.s. = not significant

**Protein**	**Antibody**	**Dilution**	**Reference**
CYP11B2 (aldosterone synthase)	Murine monoclonal antibody against CYP11B2 41-17	1:500	Gomez-Sanchez C.E., 2014 [24]
CRY1 (chriptocrome 1)	Abgent (# AP6134a)	1:100	n.a.
CRY2 (chriptocrome 2)	Abcam (# ab38872)	1:200	n.a.
HSD3B1 (type I 3β-hydroxyl-steroid dehydrogenase)	Abnova (#H00003283-M01, clone 3C11-D4)	1:1500	n.a.
HSD3B2 (type II 3β-hydroxyl-steroid dehydrogenase)	KAL-KG619	1:5000	Doi M., 2014 [13]

Supplemental Table S2. Primary antibodies used for the immunohistochemical staining and respective dilution. n.a. = not applicable.
